# Analysis of State Cannabis Laws and Dispensary Staff Recommendations to Adults Purchasing Medical Cannabis

**DOI:** 10.1001/jamanetworkopen.2021.24511

**Published:** 2021-09-15

**Authors:** Jessica S. Merlin, Andrew Althouse, Robert Feldman, Julia H. Arnsten, Hailey W. Bulls, Jane M. Liebschutz, Shannon M. Nugent, Steven R. Orris, Rebecca Rohac, Joanna L. Starrels, Benjamin J. Morasco, Devan Kansagara

**Affiliations:** 1 Challenges in Managing and Preventing Pain Clinical Research Center, University of Pittsburgh, Pittsburgh, Pennsylvania; 2 Division of General Internal Medicine, Center for Research on Health Care, University of Pittsburgh, Pittsburgh, Pennsylvania; 3 Division of General Internal Medicine, Montefiore Medical Center, Albert Einstein College of Medicine, Bronx, New York; 4 Department of Psychiatry, Oregon Health and Science University, Portland; 5 Center to Improve Veteran Involvement in Care, VA Portland Health Care System, Portland, Oregon; 6 Department of Medicine, Oregon Health and Science University, Portland

## Abstract

**Importance:**

Over the last decade, cannabis has become more accessible through the proliferation of dispensaries in states that have legalized its use. Most patients using cannabis for medical purposes report getting advice from dispensaries, yet there has been little exploration of frontline dispensary staff practices.

**Objective:**

To describe the practices of frontline dispensary workers who interact with customers purchasing cannabis for medical purposes and assess whether dispensary practices are associated with medicalization of state cannabis laws (degree to which they resemble regulation of prescription or over-the-counter drugs) and statewide adult use.

**Design, Setting, and Participants:**

This nationwide cross-sectional survey study was conducted from February 13, 2020, to October 2, 2020, using an online survey tool. Potential respondents were eligible if they reported working in a dispensary that sells tetrahydrocannabinol-containing products and interacting with customers about cannabis purchases.

**Main Outcomes and Measures:**

Participant responses to questions about formulating customer recommendations and talking to customers about risks.

**Results:**

The 434 survey responses from 351 unique dispensaries were most often completed by individuals who identified as budtenders (40%), managers (32%), and pharmacists (13%). Most respondents reported basing customer recommendations on the customer’s medical condition (74%), the experiences of other customers (70%), the customer’s prior experience with cannabis (67%), and the respondent’s personal experience (63%); fewer respondents relied on clinician input (40%), cost (45%), or inventory (12%). Most respondents routinely advised customers about safe storage and common adverse effects, but few counseled customers about cannabis use disorder, withdrawal, motor vehicle collision risk, or psychotic reactions. A higher state medicalization score was significantly associated with using employer training (odds ratio, 1.41; 95% CI, 1.18-1.67) and physician or clinician input (odds ratio, 1.23; 95% CI, 1.05-1.43) as a basis for recommendation. Medicalization score was not associated with counseling about cannabis risks.

**Conclusions and Relevance:**

This survey study provides insight into how frontline dispensary staff base cannabis recommendations and counsel about risks. The findings may have utility for clinicians to counsel patients who purchase cannabis, customers who want to be prepared for a dispensary visit, and policy makers whose decisions affect cannabis laws.

## Key Points

**Question:**

What are the self-reported practices of frontline dispensary staff who interact with customers purchasing cannabis for medical purposes?

**Findings:**

In this survey study of responses from 434 staff from 351 unique dispensaries, recommendations were most often based on experiences of the respondent, other customers, and other staff recommendations. Higher medicalization scores were associated with physician or clinician input as a basis for recommendation.

**Meaning:**

The results of this survey study suggest a need for clinicians to be aware of dispensary staff practices and to engage in discussions about the benefits and harms of cannabis use with their patients.

## Teaser

This survey study describes the practices of frontline dispensary staff who interact with customers purchasing cannabis for medical purposes and assesses whether dispensary practices vary according to state cannabis law medicalization.

## Introduction

Cannabis access has substantially increased over the past decade in the US,^1,2,3,4^ particularly with the proliferation of dispensaries in states that have legalized cannabis use. In this context, clinicians may encounter increasing numbers of patients with questions about using cannabis for medical purposes. It is not known what proportion of cannabis used is obtained from dispensaries; a 2020 study^1^ suggests that individuals obtain cannabis from dispensaries as well as from alternative sources (eg, illicit markets, home growth). However, dispensary sales are increasing, underscoring their importance in the US cannabis market.^2^ Moreover, most patients who use cannabis for medical purposes report receiving specific advice about cannabis formulations and use patterns directly from dispensaries rather than from clinicians.^3^

Understanding how dispensary staff interact with consumers is key to knowing how consumers make cannabis purchasing decisions. Calcaterra et al^4^ describe the “void in clinician counseling of cannabis use,” which they propose is due to federal laws that prohibit physicians from prescribing cannabis and lack of evidence-based recommendations about benefits and harms. The health care clinician’s role is relegated to assessing whether the patient has a state-sanctioned qualifying condition, resulting in a phenomenon the authors describe as “cannabis dispensary workers as proxy clinicians.” There has been little exploration of frontline dispensary staff practices.

One factor that complicates understanding dispensary practices is state law variability. A recent study^5^ of all states with legal medical cannabis identified substantial variability in requirements across several domains, including manufacturing or testing, product labeling, and types of products permissible for sale, as well as limits on the supply or dose that can be dispensed. The degree to which these regulations resemble regulation of prescription and over-the-counter drugs has been termed *medicalization*.^5^

This study describes the practices of frontline dispensary staff who interact with customers purchasing cannabis for medical purposes. We assessed whether dispensary practices varied according to state cannabis law medicalization and whether the state allowed adult use. We hypothesized that respondents from states with more medicalized programs vs less medicalized programs would rely on traditional sources of information (eg, clinician input, employer training) and would talk with customers more about risks.

## Methods

### Survey Development

After reviewing relevant literature^6,7,8,9,10^ and finding no detailed survey querying staff practices, our team developed a survey specifically to query dispensary staff practices. The eMethods in the Supplement presents the complete survey. The survey included questions about the basis of staff recommendations to customers; participants could choose from 18 potential bases (eg, customers’ medical conditions, experiences of other customers) and were asked to check all that applied. Participants were also asked how often they talk to customers about specific cannabis risks (eg, cannabis use disorder, motor vehicle collisions) and to answer on a scale of 1 to 5 where 1 indicates never and 5-indicates always. Other questions included sociodemographic characteristics, role or job at the dispensary, and personal experiences with cannabis. Participants were asked to think about customers seeking cannabis for medical conditions or symptoms, regardless of whether they buy cannabis using a medical marijuana card or recreationally. We specified that we were not interested in customers who use cannabis purely for recreational purposes. The survey was piloted with several dispensary industry contacts for content and understanding and was revised iteratively. This study followed the relevant portions of the American Association for Public Opinion Research (AAPOR) reporting guideline for survey studies. This study was classified as exempt from review and informed patient consent by the University of Pittsburgh Institutional Review Board because of the study type.

### Sampling Strategy and Recruitment

To identify US dispensaries, we purchased a list of 4715 dispensaries across 34 states from a marketing company. We identified additional dispensaries through internet searches of state databases and websites such as Leafly.com and WeedMaps.com. This is an accepted method of locating dispensaries that are operating at a given time,^11^ but since dispensaries open and close frequently, the true denominator of dispensaries cannot be definitively determined.

We initially planned to recruit respondents with telephone calls to a representative random sample of all identified US dispensaries. We generated random samples of 1000 dispensaries stratified by state, and research staff called these dispensaries using a standard script. The script involved asking for a manager who could send the survey to frontline staff. We found that dispensary managers were often difficult to reach, and managers and higher-level administrators expressed concern about the potentially proprietary nature of the information we wished to gather (eg, dispensary practices) and our intentions regarding speaking directly to staff (eg, poaching staff for opening of new dispensaries). This recruitment method led to few completed surveys.

To increase responses, we mailed hard copies of the survey to all identified US dispensaries including instructions for online completion, and used a snowball sampling approach by sending an electronic link to leaders at 2 large national dispensary chains and a cannabis retailers association. Although not specifically encouraged, the electronic link could be forwarded so that survey responses were not limited to the dispensaries we directly recruited. To calculate the response rate for telephone calls and mailers, responses were attributed to the most recent method of contact.

### Eligibility Criteria

Participants were eligible to complete the survey if they reported working in a dispensary and interacting with customers about cannabis product purchases. To be considered a cannabis dispensary, the dispensary had to sell tetrahydrocannabinol (THC)–containing products, whether for medical or adult-use purposes or both; we excluded stores that sold only cannabidiol products. We excluded employees under the age of 18 years and those who had been in their current position for less than 3 months.

### Survey Administration

All surveys were delivered online via Qualtrics. The survey was developed and then piloted from May 2019 to January 2020. It opened for completion by respondents on February 13, 2020, and closed on October 2, 2020. Respondents were given the choice of receiving a $10 payment card immediately on completion of the survey or the option of being randomly selected to receive a $250 payment card at the end of the study.

### State Medicalization Score and Statewide Adult Use Variables

A state cannabis medicalization score was developed based on a review of cannabis laws.^5^ This score includes 7 domain scores (patient-clinician relationship, manufacturing and testing, product labeling, types of products, supply and dose limit, prescription drug monitoring program, and dispensing practices) and a summary score for each state that had enacted medical cannabis laws as of July 2019. Herein, we used the summary scores, which range from 23 (least medicalized) to 86 (most medicalized). The statewide adult use variable was whether the state had legal adult use as of July 2019.

### Statistical Analysis

Continuous variables are presented as mean (SD); categorical variables are presented as frequencies and percentages. Multivariable regression analyses were used to estimate associations between state regulations and the 2 practices of interest: basis of recommendations and talking to customers about risks. A separate regression was performed with each basis of recommendation and risk as the outcome. For each regression, the 2 independent variables of interest were state medicalization score (scaled by 10-point increments) and statewide adult use (dichotomous). All models included the following prespecified covariates: age, role (eg, budtender, pharmacist), years working in the cannabis industry, receipt of sales commission, and level of education.

Logistic regression analyses were conducted for the basis-of-recommendation outcome variables; results are reported as odds ratios (ORs) with 95% CIs. The talking-to-customers-about-risk outcome variables were modeled using ordinary least-squares regression; results are presented as regression coefficients and standard errors. All statistical analyses were performed using R version 3.6.0 (R Project for Statistical Computing). Statistical significance was set at 2-sided *P* < .05.

Our survey was mailed and emailed widely and could have been forwarded to unanticipated respondents, and came with a small financial incentive. To ensure that our analyses included only responses that could be legitimate, the primary analytic set included surveys that were at least 95% complete, from states in which sales of THC-containing products are legal, and from respondents not affiliated with major chain pharmacies or grocery stores where THC-containing products are not sold. We conducted a sensitivity analysis in which we removed dispensaries that were not on our list to contact that did not have a website (based on a prior study suggesting that if a dispensary does not have a web presence, they may no longer be open)^11^ or whose website did not confirm sales of THC-containing products.

## Results

Of 1988 dispensaries who received at least 1 telephone call attempt, 127 (6%) returned at least 1 completed survey. Of the 4733 identified US dispensaries who received a mailer, 352 (7.4%) returned at least 1 completed survey. We received 735 total responses, of which 222 were more than 95% complete, 38 were from states in which sales of THC-containing products are not legal, and 41 were from chain pharmacies or grocery stores in which cannabis is not sold, leaving 434 eligible for the primary analysis (from 351 unique dispensaries). These 351 responding dispensaries were from states with mean (SD) medicalization scores of 46 (5) vs 40 (10) from dispensaries that did not respond, and were less often from adult use states (42%) than dispensaries that did not respond (50%). Of the 434 surveys eligible for the primary analysis, 43 did not have a website confirming THC-containing product sales, leaving 391 eligible for the sensitivity analysis (from 308 unique dispensaries). The results of the sensitivity analyses were similar to the primary analyses and are presented as Supplement material (eTables 1-5, eFigure 2 in the Supplement).

The largest number of surveys were from New York, Oregon, California, and Florida (eFigure 1 in the Supplement); about one-third were from states in which adult use is legal. Surveys were most often completed by individuals who self-identified as budtenders (40%), followed by managers (32%), pharmacists (13%), and physicians, nurse practitioners, or physician assistants (5%) (Table 1). Half of the respondents reported working in the industry for more than 2 years, and 40% reported working in their current position for 2 or more years. Most respondents (88%) had completed at least some college, including 17% who completed at least some graduate school. Fifteen percent reported receiving a sales commission. Nearly two-thirds of respondents reported having a medical cannabis card, and nearly two-thirds reported using cannabis multiple times per week or daily or almost daily. Nearly half reported using cannabis for both medical and recreational purposes. More than three-quarters of respondents (78%) agreed or strongly agreed that personal cannabis use helped them advise customers.

**Table 1.** Participant Demographic Characteristics

Characteristic	No. (%)
No.	434
Age, mean (SD), y	33.4 (9.83)
Role	
Budtender	173 (39.9)
Manager	140 (32.3)
Physician/NP/PA	22 (5.1)
Pharmacist	57 (13.1)
Other	41 (9.4)
No response	1 (0.2)
Participant has medical cannabis card	
No	153 (35.3)
Yes	279 (64.3)
No response	2 (0.5)
How often participant used cannabis in past 3 mo	
Never	66 (15.2)
Multiple times	
Per year	35 (8.1)
Per month	55 (12.7)
Per week	43 (9.9)
Daily or almost daily	234 (53.9)
No response	1 (0.2)
For what purpose	
I do not use cannabis	6 (1.4)
Only for medical purposes	106 (24.4)
Only for recreational purposes	49 (11.3)
For both medical and recreational purposes	211 (48.6)
Other	61 (14.1)
No response	1 (0.2)
Personal use helps advise customers	
1 (strongly disagree)	21 (4.8)
2	13 (3)
3	53 (12.2)
4	79 (18.2)
5 (strongly agree)	260 (59.9)
No response	8 (1.8)
Years working in cannabis industry	
<6 mo	25 (5.8)
6 mo to 1 y	68 (15.7)
>1-2 y	116 (26.7)
>2 y	219 (50.5)
No response	6 (1.4)
Length of time in current position	
<6 mo	26 (6)
6 mo to 1 y	117 (27)
>1-2 y	117 (27)
>2 y	173 (39.9)
No response	1 (0.2)
Sales commission	
No	366 (84.3)
Yes	66 (15.2)
No response	2 (0.5)
Education	
Completed high school/GED or less	47 (10.8)
Some college or associates degree	181 (41.7)
Completed 4-y college degree	129 (29.7)
Some graduate school	22 (5.1)
Completed graduate school	51 (11.8)
Prefer not to answer	3 (0.7)
No response	1 (0.2)
Sex	
Male	196 (45.2)
Female	225 (51.8)
Other^a^/no response	13 (3)
State medicalization score, mean (SD)	47.56 (15.65)
Statewide adult use	
No	271 (62.4)
Yes	163 (37.6)

Abbreviations: GED, General Education Development; NP, nurse practitioner; PA, physician assistant.

^a^ The classification as “other” was taken from database/survey with no further nonbinary breakdown available.

Table 2 summarizes the bases that dispensary staff reported using to make recommendations to customers. The mean (SD) number of bases of recommendation endorsed was 9.1 (4.6). The most common responses were the customer’s medical condition (74%) and experiences of other customers (70%), followed by the customer’s prior experience with cannabis (67%), customer preference (66%), preferred time of day or night for consumption (65%), the respondent’s personal experience (63%), employer training (61%), other staff recommendations (56%), and product availability (50%).

**Table 2.** Self-report of Basis of Recommendations

Basis of recommendation	Yes response, No. (%)
Customer’s medical condition(s)	319 (73.5)
Experiences of other customers	305 (70.3)
Customer’s prior experience with cannabis	292 (67.3)
Customer preference	286 (65.9)
Daytime or nighttime consumption	283 (65.2)
Scientific articles (eg, articles from medical journals)	279 (64.3)
Your personal experience	274 (63.1)
Training provided by your employer	265 (61.1)
Other staff recommendations	242 (55.8)
Product availability	215 (49.5)
Cost	197 (45.4)
Experience of friends or colleagues	194 (44.7)
Trade literature (eg, trade magazines or websites)	191 (44.0)
Physician/clinician input	175 (40.3)
App or website that helps with product selection (eg, Strainpaint)	140 (32.3)
Product smell	127 (29.3)
Product appearance (for flower)	123 (28.3)
What needs to get moved out of inventory	52 (12.0)

Regression analyses were conducted to assess the association between state medicalization score (per 10-point increase on the scale) and statewide adult use (yes) and respondent’s use of a given basis for recommendations to customers (Table 3). A higher state medicalization score was positively associated with use of employer training (OR, 1.41; 95% CI, 1.18-1.67; *P* < .001) and physician or clinician input (OR, 1.23; 95% CI, 1.05-1.43; *P* = .01) as a basis for recommendation, and was negatively associated with using product appearance (OR, 0.78; 95% CI, 0.64-0.96; *P* = .02), the respondent’s personal experience (OR, 0.82; 95% CI, 0.69-0.98; *P* = .03), or what needs to get moved out of inventory (OR, 0.72; 95% CI, 0.55, 0.93; *P* = .01) as a basis for customer recommendations. Statewide adult use was associated with using trade literature (OR, 1.69; 95% CI, 1.05-2.75: *P* = .03), app or website (OR, 1.69; 95% CI, 1.02-2.79: *P* = .04), experience of friends or colleagues (OR, 2.09; 95% CI, 1.27-3.43; *P* < .001), product appearance (OR, 2.63; 95% CI, 1.53-4.52; *P* < .001) and product smell (OR, 3.18; 95% CI, 1.86-5.43; *P* < .001) as a basis for customer recommendations.

**Table 3.** Association of Basis of Recommendations With State Medicalization Score and Statewide Adult Use^a^

Potential basis	State medicalization score (per 10-point increment)		Statewide adult use	
	OR (95% CI)	*P* value	OR (95% CI)	*P* value
Training provided by your employer	1.41 (1.18-1.67)	<.001	1.51 (0.90-2.54)	.12
Trade literature (eg, trade magazines or websites)	0.99 (0.85-1.15)	.86	1.69 (1.05-2.75)	.03
App or website that helps with product selection (eg, Strainpaint)	0.88 (0.75-1.04)	.14	1.69 (1.02-2.79)	.04
Scientific articles (eg, articles from medical journals)	0.89 (0.76-1.04)	.16	1.42 (0.84-2.41)	.19
Physician/clinician input	1.23 (1.05-1.43)	.01	0.93 (0.57-1.53)	.78
Customer’s medical condition(s)	1.01 (0.85-1.20)	.93	0.87 (0.49-1.54)	.64
Cost	1.05 (0.90-1.21)	.55	1.0 (0.62-1.62)	.99
Product availability	0.91 (0.78-1.06)	.22	1.05 (0.65-1.69)	.85
What needs to get moved out of inventory	0.72 (0.55-0.93)	.01	1.09 (0.54-2.18)	.82
Experiences of other customers	1.04 (0.88-1.24)	.65	1.54 (0.86-2.76)	.14
Your personal experience	0.82 (0.69-0.98)	.03	1.23 (0.67-2.26)	.5
Other staff recommendations	0.86 (0.73-1.01)	.07	1.37 (0.79-2.36)	.26
Customer preference	1.05 (0.88-1.24)	.6	1.12 (0.64-1.96)	.71
Experience of friends or colleagues	0.92 (0.79-1.08)	.29	2.09 (1.27-3.43)	.004
Customer’s prior experience with cannabis	1.02 (0.85-1.22)	.86	1.65 (0.9-3.04)	.11
Daytime or nighttime consumption	1.0 (0.82-1.20)	.97	1.76 (0.93-3.34)	.08
Product smell	0.89 (0.74-1.08)	.23	3.18 (1.86-5.43)	<.001
Product appearance (for flower)	0.78 (0.64-0.956)	.02	2.63 (1.53-4.52)	<.001

Abbreviation: OR, odds ratio.

^a^ This table presents a series of logistic regression models in which each row represents the dependent variable with each column representing an independent variable in separate logistic regression models. For example, the second column of the second row indicates that a 10-point increase in the state medicalization score is associated with 1.41 times higher odds of the respondent saying that they use training provided by their employer as basis for recommendations.

Table 4 summarizes how often respondents reported talking to customers about risks. Respondents reported (on a scale of 1 to 5 where 1 indicates never and 5 indicates always) addressing potential cannabis adverse effects (response of 5: 163 [37.6%]) and safe storage practices (response of 5: 183 [42.2%]) most frequently. Development of cannabis use disorder (response of 5: 23 [5.3%]), cannabis withdrawal (response of 5: 22 [5.1%]), and psychotic reaction (response of 5: 55 [12.7%]) were reported to be addressed less frequently.

**Table 4.** How Often Respondent Talks to Customers About Risk

Risk	Response, No. (%)				
	1 (Never)	2	3	4	5 (Always)
Cannabis use disorder/addiction	120 (27.6)	120 (27.6)	110 (25.3)	60 (13.8)	23 (5.3)
Motor vehicle collisions/safe driving	65 (15)	109 (25.1)	112 (25.8)	82 (18.9)	65 (15)
Cannabis withdrawal symptoms	140 (32.3)	124 (28.6)	87 (20)	60 (13.8)	22 (5.1)
Psychotic reaction	102 (23.5)	95 (21.9)	110 (25.3)	71 (16.4)	55 (12.7)
Cannabis medication interactions	60 (13.8)	83 (19.1)	100 (23)	102 (23.5)	88 (20.3)
Potential cannabis adverse effects (eg, sleepiness, paranoia)	15 (3.5)	48 (11.1)	82 (18.9)	124 (28.6)	163 (37.6)
Safe storage away from children and pets	19 (4.4)	46 (10.6)	85 (19.6)	100 (23)	183 (42.2)

Table 5 presents results from regression analyses that assessed variables associated with counseling about cannabis risks. Statewide adult use was associated with a small increase in counseling regarding safe storage away from children and pets (B = 0.3; SE = 0.13; *P* = .03). Otherwise state medicalization score and adult use were not associated with counseling about cannabis risks.

**Table 5.** Association of Talking to Customers About Risk With State Medicalization Score and Statewide Adult Use^a^

Risk	State medicalization score (per 10-point increment)		Statewide adult use	
	B (SE)	*P* value	B (SE)	*P* value
Cannabis use disorder/addiction	0.003 (0.04)	.95	−0.2 (0.13)	.12
Motor vehicle collisions/safe driving	0.02 (0.05)	.61	0.04 (0.15)	.80
Cannabis withdrawal symptoms	−0.04 (0.04)	.34	−0.21 (0.13)	.10
Psychotic reaction	0.03 (0.06)	.51	−0.02 (0.07)	.79
Cannabis medication interactions	0.08 (0.05)	.09	−0.16 (0.15)	.3
Potential cannabis adverse effects (eg, sleepiness, paranoia)	0.01 (0.04)	.75	−0.17 (0.13)	.18
Safe storage away from children and pets	0.04 (0.04)	.36	0.3 (0.13)	.03

^a^ This table presents a series of linear regression models in which each row represents the dependent variable with each column representing an independent variable in separate linear regression models. For example, the top-left-hand cell indicates that a 10-point increase in the state medicalization score is associated with a mean change of 0.003 in the scale response to how often the respondent talks to their customers about the risks of cannabis use disorder/addiction.

## Discussion

In this national survey study, most dispensary staff had worked in the cannabis industry for 1 or more years, were college-educated, and many used cannabis for medical or adult-use purposes. Staff often relied on personal and coworker experience to make recommendations. While most staff reported routinely counseling customers about safe storage of cannabis and routine cannabis adverse effects such as sleepiness, few reported routinely counseling customers about cannabis-related risks such as psychosis, motor vehicle collisions, cannabis withdrawal syndrome, or cannabis use disorder.

State medicalization and adult use were associated with how respondents based their recommendations. Being in a state with a higher medicalization score was associated with an increased likelihood of using employer training and physician or clinician input as a basis for clinical recommendations. This finding may indicate that medicalization is associated with an environment where physician or clinician input is more likely to be incorporated. Additionally, respondents who lived in states with legalized adult and medical use were more likely to endorse using personal experience and cannabis product smell or appearance as a basis for recommendations, perhaps indicating more product familiarity. However, state medicalization and adult use were generally not associated with counseling about cannabis-related risks. Although cannabis risks have been well-characterized, our findings suggest that state regulations have not been associated with dispensaries where such risks are emphasized.

It could be expected that dispensary workers do not routinely counsel customers about risks. A survey of the US general population found that less than half of respondents who had reported any past year cannabis use were concerned about risks such as cannabis use disorder, and that perceived risk of regular cannabis use has decreased over time.^10,12^ To our knowledge, no current research clearly outlines the balance of cannabis benefits and harms. Despite customers’ potential reliance on dispensaries for health-related information about cannabis,^4^ it may not be reasonable to expect dispensaries, which are retail and not medical establishments, to bear primary responsibility for such counseling, much as alcohol retailers may not provide counseling about alcohol-related harms.

Clinicians may not be aware of dispensary staff practices, and engagement in discussions about the benefits and harms of cannabis use with their patients is warranted. For example, clinicians might alert patients that dispensary staff purchasing recommendations may be based on nonprofessional anecdotal experience and they should not expect counseling about harms. This approach could be an opportunity for the health care clinician to have evidence-based discussions with their patients about cannabis^13^ including reviewing cannabis-related harms, and to counsel patients about potential ways to mitigate these harms, such as previously-published guidance for low-risk cannabis use.^14^

Although a higher degree of medicalization was associated with using health care clinician input and information from training, legal statewide adult use was associated with using personal experiences of friends or colleagues. Additionally, as research^5^ has shown, there is substantial variability in states’ degree of medicalization. Therefore, we suggest that dispensary environments are highly variable and cannot be assumed to be medical environments. Certifying clinicians, particularly in less medicalized states, should be aware that the decision-making that occurs at a dispensary is often different from that which occurs in a medical environment such as a pharmacy.

There is likely a gap between the way cannabis is perceived by dispensary staff and the way it is perceived by clinicians. Our findings suggest that dispensary staff are comfortable giving advice from an experiential standpoint. Conversely, clinicians may view cannabis through a traditional pharmacotherapeutic lens and be troubled at the lack of standardized dosing, regulatory oversight,^15^ and the uncertainties in the evidence base, leading to a low comfort level related to recommending medical cannabis.^8^

### Strengths and Limitations

This study has strengths. To our knowledge, this is the first national study to examine self-reported dispensary staff practices. A prior study^6^ reported results from a smaller sample of dispensary workers in 2 cities but focused on comparing characteristics and practice among workers who had and had not received training and on their online behaviors. The procedural revisions we made to our initial recruitment approach could help advance knowledge in the field about dispensary staff research recruitment methods. We attempted to call a large number of randomly selected dispensaries from a comprehensive list. However, dispensary lists may be obsolete due to dispensaries opening and closing, such that a denominator for response rates cannot be determined. Given the importance of research in the industry, we recommend that metrics assess the use and success of a best possible approach. Such metrics could include reach of a survey across states in which cannabis is legal and the absolute number of responses. In this case, we had hundreds of responses from most states in which cannabis is legal, suggesting that this approach is viable for addressing frontline dispensary practices.

This study also has limitations. Responses are based on self-report; actual staff practices are unknown. Additionally, the degree to which the respondents are representative of dispensary workers nationally, or the responses are representative of dispensary practices nationally, is not known. Respondents may be fundamentally different from nonrespondents in unmeasured ways that could confound findings or limit generalizability. Our sample size is fairly modest, limiting statistical power to detect small effects. Finally, the field is dynamic: dispensaries open and close and laws and policies change.

## Conclusions

This survey study provides insight into frontline dispensary staff cannabis recommendations and counsel about risks. Our findings may have utility for clinicians to counsel patients who purchase cannabis from dispensaries, customers who want to be prepared for a dispensary visit, and policy makers whose decisions affect state cannabis laws.

## Notes

Supplement. **eMethods.** Qualtrics Survey Distributed for this Study **eTable 1.** Sensitivity Analysis (N = 391): Demographics **eTable 2.** Sensitivity Analysis (N = 391): Self-report of Basis of Recommendations **eTable 3.** Sensitivity Analysis (N = 391): Association of Basis of Recommendations With State Medicalization Score and Statewide Adult Use **eTable 4.** Sensitivity Analysis (N = 391): How Often Respondent Talks to Customers About Risk **eTable 5.** Sensitivity Analysis (N = 391): Association of Talking to Customers About Risk With State Medicalization Score and Statewide Adult Use **eFigure 1.** Survey Response by State, Primary Analysis, N = 434 **eFigure 2.** Sensitivity Analysis: Survey Response by State, N = 391
