# Supplementary material for: Analysis of State Cannabis Laws and Dispensary Staff Recommendations to Adults Purchasing Medical Cannabis
Source: JAMA Netw Open. 2021 Sep 15;4(9):e2124511. doi: 10.1001/jamanetworkopen.2021.24511 (PMC8444019; doi:10.1001/jamanetworkopen.2021.24511)
Supplement: Supplement. — eMethods. Qualtrics Survey Distributed for this Study eTable 1. Sensitivity Analysis (N = 391): Demographics eTable 2. Sensitivity Analysis (N = 391): Self-report of Basis of Recommendations eTable 3. Sensitivity Analysis (N = 391): Association of Basis of Recommendations With State Medicalization Score and Statewide Adult Use eTable 4. Sensitivity Analysis (N = 391): How Often Respondent Talks to Customers About Risk eTable 5. Sensitivity Analysis (N = 391): Association of Talking to Customers About Risk With State Medicalization Score and Statewide Adult Use eFigure 1. Survey Response by State, Primary Analysis, N = 434 eFigure 2. Sensitivity Analysis: Survey Response by State, N = 391 [file jamanetwopen-e2124511-s001.pdf]

Supplemental Online Content

Merlin JS, Althouse A, Feldman R, et al. Analysis of state cannabis laws and dispensary staff recommendations to adults purchasing medical cannabis. *JAMA Netw Open*. 2021;4(9):e2124511. doi:10.1001/jamanetworkopen.2021.24511

eMethods. Qualtrics Survey Distributed for this Study
eTable 1. Sensitivity Analysis (N=391): Demographics
eTable 2. Sensitivity Analysis (N=391): Self-report of Basis of Recommendations
eTable 3. Sensitivity Analysis (N=391): Association of Basis of Recommendations With State Medicalization Score and Statewide Adult Use
eTable 4. Sensitivity Analysis (N=391): How Often Respondent Talks to Customers About Risk
eTable 5. Sensitivity Analysis (N=391): Association of Talking to Customers About Risk With State Medicalization Score and Statewide Adult Use
eFigure 1. Survey Response by State, Primary Analysis, N=434
eFigure 2. Sensitivity Analysis: Survey Response by State, N=391

This supplemental material has been provided by the authors to give readers additional information about their work.

© 2021 Merlin S et al. *JAMA Network Open.*

eMethods. Qualtrics survey distributed for this study

Q1. Do you work in a place where cannabis is sold?
Please note: We define cannabis as some combination of THC and CBD-based products (**NOT** CBD-only)

- Yes
- No

Q2. Have you worked in your current position for at least 3 months?

- Yes
- No

Q3. Do you educate, counsel, or otherwise help customers make decisions about cannabis?

- Yes
- No

Q4. Are you 18 years of age or older?

- Yes
- No

Your Dispensary Information

Q5. What is the name of your dispensary? This is for tracking purposes only. Your responses will not be shared with any other dispensary staff. If you work at more than one dispensary, please list the dispensary where you work the most hours.

Q6. What is the zip code of the dispensary where you work?

Attitudes About Cannabis Benefits and Risks
In the following questions, we will ask for your opinion on the helpfulness of cannabis for certain symptoms and medical conditions. We recognize that there are many different types of cannabis that can be helpful for different purposes. When answering these questions, please consider all types of cannabis available in your dispensary.

Q7. On average, how helpful do you think cannabis is for each of the following symptoms?

- Chronic pain: *Not very helpful (1), (2), (3), (4), Very helpful (5)*
- Headache: *Not very helpful (1), (2), (3), (4), Very helpful (5)*
- Muscle spasms: *Not very helpful (1), (2), (3), (4), Very helpful (5)*
- Poor appetite/weight loss: *Not very helpful (1), (2), (3), (4), Very helpful (5)*
- Nausea/Vomiting: *Not very helpful (1), (2), (3), (4), Very helpful (5)*
- Insomnia: *Not very helpful (1), (2), (3), (4), Very helpful (5)*
- Anxiety: *Not very helpful (1), (2), (3), (4), Very helpful (5)*
- Stress: *Not very helpful (1), (2), (3), (4), Very helpful (5)*
- depression: *Not very helpful (1), (2), (3), (4), Very helpful (5)*
- Optional: other symptom, please specify

Q8. On average, how helpful do you think cannabis is for each of the following medical conditions?

- Epilepsy (Seizures): *Not very helpful (1), (2), (3), (4), Very helpful (5)*
- HIV: *Not very helpful (1), (2), (3), (4), Very helpful (5)*
- Glaucoma: *Not very helpful (1), (2), (3), (4), Very helpful (5)*
- Cancer: *Not very helpful (1), (2), (3), (4), Very helpful (5)*
- Inflammatory Bowel Disease (e.g., Ulcerative Colitis, Crohn's Disease): *Not very helpful (1), (2), (3), (4), Very helpful (5)*
- Post-Traumatic Stress Disorder (PTSD): *Not very helpful (1), (2), (3), (4), Very helpful (5)*
- Autism: *Not very helpful (1), (2), (3), (4), Very helpful (5)*

© 2021 Merlin S et al. *JAMA Network Open.*

- Hepatitis C: *Not very helpful (1), (2), (3), (4), Very helpful (5)*
- Amyotrphic Lateral Sclerosis: *Not very helpful (1), (2), (3), (4), Very helpful (5)*
- Tourette's Syndrome: *Not very helpful (1), (2), (3), (4), Very helpful (5)*
- Alzheimer's Disease: *Not very helpful (1), (2), (3), (4), Very helpful (5)*
- Parkinson's Disease: *Not very helpful (1), (2), (3), (4), Very helpful (5)*
- Sickle Cell Disease: *Not very helpful (1), (2), (3), (4), Very helpful (5)*
- Psoriasis: *Not very helpful (1), (2), (3), (4), Very helpful (5)*
- Cerebral Palsy: *Not very helpful (1), (2), (3), (4), Very helpful (5)*
- Cystic Fibrosis: *Not very helpful (1), (2), (3), (4), Very helpful (5)*
- End-of-life: *Not very helpful (1), (2), (3), (4), Very helpful (5)*
- Diabetes: *Not very helpful (1), (2), (3), (4), Very helpful (5)*
- Obstructive Sleep Apnea: *Not very helpful (1), (2), (3), (4), Very helpful (5)*
- End-stage Kidney Disease (patients on hemodialysis): *Not very helpful (1), (2), (3), (4), Very helpful (5)*
- Optional: Other medical condition, please specify

Q9. How worried are you about the following potential negative effects of cannabis for your customers?

- Interactions with medications: *Not at all worried (1), (2), (3), (4), Very worried (5)*
- Legal problems: *Not at all worried (1), (2), (3), (4), Very worried (5)*
- Increase in stress: *Not at all worried (1), (2), (3), (4), Very worried (5)*
- Anxiety: *Not at all worried (1), (2), (3), (4), Very worried (5)*
- Depression: *Not at all worried (1), (2), (3), (4), Very worried (5)*
- Decreased energy: *Not at all worried (1), (2), (3), (4), Very worried (5)*
- Disrupted sleep: *Not at all worried (1), (2), (3), (4), Very worried (5)*
- New or worsening health problems: *Not at all worried (1), (2), (3), (4), Very worried (5)*
- Impaired memory: *Not at all worried (1), (2), (3), (4), Very worried (5)*
- Delayed reaction time: *Not at all worried (1), (2), (3), (4), Very worried (5)*
- Decrease in intelligence (IQ): *Not at all worried (1), (2), (3), (4), Very worried (5)*
- Personal or relationship problems: *Not at all worried (1), (2), (3), (4), Very worried (5)*
- Safety risks such as motor vehicle or other accidents: *Not at all worried (1), (2), (3), (4), Very worried (5)*
- Risk of developing cannabis use disorder/addiction: *Not at all worried (1), (2), (3), (4), Very worried (5)*
- Increased use of illicit drugs: *Not at all worried (1), (2), (3), (4), Very worried (5)*
- Cost: *Not at all worried (1), (2), (3), (4), Very worried (5)*
- Judgement from physicians or other healthcare professionals: *Not at all worried (1), (2), (3), (4), Very worried (5)*
- Judgement from family/friends: *Not at all worried (1), (2), (3), (4), Very worried (5)*
- Optional: other potential negative effect, please specify

Q10. How often do you talk with customers about:

- Cannabis use disorder/addiction: *Never (1), (2), (3), (4), Always (5)*
- Motor vehicle accidents/safe driving: *Never (1), (2), (3), (4), Always (5)*
- Cannabis withdrawal symptoms: *Never (1), (2), (3), (4), Always (5)*
- Psychotic reaction: *Never (1), (2), (3), (4), Always (5)*
- Cannabis-medication interactions: *Never (1), (2), (3), (4), Always (5)*
- Potential cannabis side effects (e.g., sleepiness, paranoia): *Never (1), (2), (3), (4), Always (5)*
- Safe storage away from children and pets: *Never (1), (2), (3), (4), Always (5)*

Q11. How safe is it for pregnant women to use cannabis? *Completely unsafe (1), (2), (3), (4), Completely safe (5), I don't know (0)*

© 2021 Merlin S et al. *JAMA Network Open.*

Q12. How safe is it for older adults (age >65) to use cannabis? *Completely unsafe (1), (2), (3), (4), Completely safe (5), I don't know (0)*

Observational About Customers' Cannabis Use and Attitudes

Q13. According to your own observations, how often do the following occur:

- Customers are generally enthusiastic about trying cannabis: *Never (1), (2), (3), (4), Always (5), I don't know (0)*
- Customers are generally hesitant to try cannabis: *Never (1), (2), (3), (4), Always (5), I don't know (0)*
- Customers have a formulation (e.g., vape vs. edible) preference for their cannabis: *Never (1), (2), (3), (4), Always (5), I don't know (0)*
- Customers have a dose preference for their cannabis: *Never (1), (2), (3), (4), Always (5), I don't know (0)*
- Customers seek cannabis for help with a medical (physical or mental) condition: *Never (1), (2), (3), (4), Always (5), I don't know (0)*
- Customers use cannabis to reduce use of prescribed medications: *Never (1), (2), (3), (4), Always (5), I don't know (0)*
- Customers use cannabis to replace prescribed medications: *Never (1), (2), (3), (4), Always (5), I don't know (0)*
- Customers seek cannabis for its pleasurable effects (e.g., to get high) : *Never (1), (2), (3), (4), Always (5), I don't know (0)*
- Cost makes it difficult for my customers to purchase cannabis: *Never (1), (2), (3), (4), Always (5), I don't know (0)*

Q14. What do you do when you encounter a customer who you suspect has a cannabis use disorder (also known as cannabis addiction)? Check all that apply:

- Refer to a physician or other health professional
- Discuss purchasing cannabis products that may help with the cannabis use disorder
- I do not do anything differently for such customers
- I have never encountered a customer who I suspected had cannabis use disorder
- I have never heard of cannabis use disorder
- I do not believe cannabis is addictive
- Other, please describe

Q15. What do you do when you encounter a customer who is using cannabis to treat a medical condition (such as cancer, HIV/AIDS, multiple sclerosis)? Check all that apply:

- Encourage customer to seek traditional medical care in addition to cannabis
- Encourage customer to seek traditional medical care instead of cannabis
- Encourage customer to inform physician or other healthcare professional about cannabis use
- Encourage customer to continue only cannabis
- Encourage customer to do additional research online
- Other, please describe
- I don't do anything differently
- I have never encountered a customer who is using cannabis to treat a medical condition

© 2021 Merlin S et al. *JAMA Network Open.*

Q16. What do you do when you encounter a customer who is using cannabis to treat depression, anxiety, or post-traumatic stress disorder? Check all that apply:

- Encourage customer to seek traditional medical/mental health care in addition to cannabis
- Encourage customer to seek traditional medical/mental health care instead of cannabis
- Encourage customer to inform physician or other healthcare professional about cannabis use
- Encourage customer to do additional research online
- Other, please describe
- I don't do anything differently
- I have never encountered a customer who us using cannabis to treat one of these disorders

Q17. What do you do when you encounter a customer who is using cannabis to treat a serious mental illness (such as schizophrenia, bipolar disorder, psychosis)? Check all that apply:

- Encourage customer to seek traditional medical/mental health care in addition to cannabis
- Encourage customer to seek traditional medical/mental health care instead of cannabis
- Encourage customer to inform physician or other healthcare professional about cannabis use
- Encourage customer to do additional research online
- Other, please describe
- I don't do anything differently
- I have never encountered a customer who is using cannabis to treat a serious mental illness

Q18. What do you do when you encounter a customer who is using cannabis to treat an opioid use disorder (addiction to opioid medications)? Check all that apply:

- Encourage customer to seek traditional medical/mental health care in addition to cannabis
- Encourage customer to seek traditional medical/mental health care instead of cannabis
- Encourage customer to inform physician or other healthcare professional about cannabis use
- Encourage customer to do additional research online
- Other, please describe
- I don't do anything differently
- I have never encountered a customer who is using cannabis to treat an opioid use disorder

Q19. Have you ever advised a customer NOT to purchase ANY cannabis products?

- Yes
- No

Q20. Select all reasons why you have advised against cannabis purchase. Check all that apply:

- Pregnancy or nursing
- Depression
- Anxiety
- Post-traumatic stress disorder
- Serious mental illness (schizophrenia, bipolar disorder, psychosis)
- Cognitive impairment (e.g., dementia)
- Customer was an older adult (age >65)
- Customer having difficulty affording cannabis
- Customer having legal problems related to cannabis
- Customer has withdrawal symptoms
- Customer needs more cannabis for the same effect
- Customer having difficulty keeping a job
- Customer having relationship problem (e.g., with partner or other close family/friends)
- Customer appeared intoxicated
- Other, please describe

© 2021 Merlin S et al. *JAMA Network Open.*

Q21. Does your dispensary have protocols for addressing the special needs of any of the following groups? Check all that apply:

- Pregnant or nursing women
- People with schizophrenia
- People with cognitive impairment (e.g., dementia)
- Older adults (>65)
- My dispensary does not have protocols for addressing any of these groups

Q22. How comfortable are you talking to customers about cannabis use to manage medical conditions and symptoms (e.g., pain, nausea, appetite, sleep)? *Very uncomfortable (1), (2), (3), (4), Very comfortable (5)*

Customer Advice

For the next questions, we will ask you about how you make recommendations about which product to purchase. If your dispensary sells both recreational and medical cannabis, please think about both recreational and medical customers when answering these questions.

Q23. On what do you base your cannabis recommendations in terms of dose, strain, and route of administration (e.g. vape, tincture, etc.)? Check all that apply:

- Training provided by your employer
- Scientific articles (e.g., articles from medical journals)
- Trade literature (e.g., trade magazines or websites)
- App or website that helps with product selection (e.g., Strainprint)
- Experiences of other customers
- Customer's medical condition(s)
- Product availability
- What needs to get moved out of inventory
- Physician/clinician input
- Your personal experience
- Other staff recommendations
- Customer preference
- Experience of friends or colleagues
- Customer’s prior experience with cannabis
- Cost
- Product smell
- Product appearance (for flower)
- Daytime or nighttime consumption
- Other, please describe
- I do not make recommendations on dose

Q24. How often do you recommend the use of more than one product to a customer? *Never (1), (2), (3), (4), Always (5)*

Q25. If you provide education/resources to your customers on the products they purchase, what does that education involve? Check all that apply:

- No education/resources provided
- Handouts
- Website (please list)
- Smartphone apps (please list)
- Support groups
- Online forums
- Personal experience
- Other, please describe

Q26. Does your dispensary sell both recreational and medical cannabis?

- Yes
- No

Q27. How do you decide whether to recommend medical or recreational cannabis for an individual customer?

<u>Questions About Customers</u>
The next few questions will ask about your customers.

Q28. I interact with my customers' physicians and/or healthcare providers: *Never (1), (2), (3), (4), Always (5)*

Q29. Among customers you interact with during at least two visits, how do you check if cannabis is working for them? Check all that apply:

- I do not monitor
- Use of standardized questionnaires (please list)
- Smartphone apps (please list)
- From conversations with the patient
- Physician/clinician input
- My general sense that the product is working or not working
- Other, please describe

Q30. Among customers you interact with during at least two visits, how often do you ask about cannabis side effects? *Never (1), (2), (3), (4), Always (5)*

Q31. What are some reasons you might change a customer's cannabis regimen? Check all that apply:

- I don't usually make changes
- Based on the results of monitoring whether cannabis is working for them
- Based on results of monitoring side effects
- Based on customer request
- Based on physician/clinician input
- Cost
- Other, please describe

Q32. In your experience how much does the average medical marijuana customer spend on a month's supply of cannabis or cannabis-related products (e.g. vape pen)?

Q33. In your experience how much does the average recreational marijuana customer spend in dollars on a month's supply of cannabis or cannabis-related products (e.g. vape pen)?

<u>Questions About Your Job</u>
The following questions relate to your job. If you work in more than one dispensary, please answer all questions for the dispensary you listed at the beginning of the survey.

Q34. In your cannabis-related job, are you:

- Full time
- Part-time

Q35. How long have you been in your current cannabis related position?

- Less than 6 months
- 6 months- 1 year
- >1-2 years
- More than 2 years

© 2021 Merlin S et al. *JAMA Network Open.*

Q36. How long have you worked in the cannabis industry?

Q37. How much do you earn in your cannabis-related job?
- Hourly wage
- Weekly salary
- Monthly salary
- Prefer not to answer

Q38. Do you receive sales commission?
- Yes
- No

Q39. What % of your sales is commission?

Q40. What types of sales are included in your commission? Check all that apply:
- Cannabis products
- Clothing
- Paraphernalia
- Other, please describe

Q41. The following best describes my role:
- Person who gives advice to customers on marijuana (known as a "budtender" in some states)
- Manager
- Physician
- Nurse practitioner
- Physician assistant
- Pharmacist
- Other, please describe

Q42. I work in an environment that sells the following (check all that apply):
- Cannabis for medical purposes only
- Cannabis for recreational purposes only
- Cannabis for recreational and medical purposes
- CBD-only products
- Paraphernalia (e.g., vaping pens)
- Clothing
- Other, please describe

Q43. How familiar are you with current cannabis laws in your state? *Very unfamiliar (1), (2), (3), (4), Very familiar (5)*

<u>Personal Use of Cannabis</u>
The following questions ask about your use of cannabis. As a reminder, we define cannabis as some combination of THC and CBD-based products (NOT CBD-only)

Q44. In the past three months, how often have you used cannabis?
- Never
- Multiple times per year
- Multiple times per month
- Multiple times per week
- Daily or almost daily

© 2021 Merlin S et al. *JAMA Network Open.*

Q45. For what purpose do you use cannabis?

- Only for medical purposes
- Only for recreational purposes
- For both medical and recreational purposes
- Other, please describe
- I do not use cannabis

Q46. Do you have a valid medical cannabis card?

- Yes
- No

Q47. My personal experience with cannabis helps me to do my job better. *Strongly disagree (1), (2), (3), (4), Strongly agree (5)*

Demographics

Q48. What is your age?

Q49. What is your gender?

- Male
- Female
- Non-binary/Third gender
- Prefer not to answer

Q50. Are you of Hispanic, Latino, or Spanish origin?

- Yes
- No
- Prefer not to answer

Q51. How would you describe yourself? Check all that apply:

- American Indian or Alaska Native
- Asian
- Black or African-American
- Native Hawaiian or Other Pacific Islander
- White
- Other
- Prefer not to answer

Q52. What is your highest level of education?

- Have not completed high school
- High school graduate or GED
- Some college or Associates Degree
- Completed 4-year college degree
- Some graduate school
- Completed graduate school
- Prefer not to answer

Open-ended Questions

Q53. What, if anything, would help you and your colleagues in providing advice about the medical use of cannabis to your customers?

Q54. Can you describe how COVID-19 has affected your work?

© 2021 Merlin S et al. *JAMA Network Open.*

eTable 1. Sensitivity Analysis (N=391): Demographics

**Age**	
Age (Q16)	33.5 ± 10.1
**Role**	
Budtender	163 (41.7%)
Manager	137 (35%)
Physician/NP/PA	12 (3.1%)
Pharmacist	38 (9.7%)
Other, Please Describe	40 (10.2%)
No Response	1 (0.3%)
**Participant has medical cannabis card**	
No	141 (36.1%)
Yes	248 (63.4%)
No Response	2 (0.5%)
**How often participant uses cannabis in past 3 months**	
Never	53 (13.6%)
Multiple times per year	18 (4.6%)
Multiple times per month	45 (11.5%)
Multiple times per week	42 (10.7%)
Daily or almost daily	232 (59.3%)
No Response	1 (0.3%)
**For what purpose**	
I do not use cannabis	6 (1.5%)
Only for medical purposes	101 (25.8%)
Only for recreational purposes	39 (10%)
For both medical & recreational purposes	194 (49.6%)
Other, please describe	50 (12.8%)
No Response	1 (0.3%)
**Personal use helps advise customers**	
1 - Strongly Disagree	20 (5.1%)
2	7 (1.8%)
3	38 (9.7%)
4	64 (16.4%)
5 - Strongly Agree	254 (65%)
No Response	8 (2%)
**Years working in cannabis industry**	
Less Than 6 Months	23 (5.9%)
6 Months-1 Year	68 (17.4%)
1-2 Years	105 (26.9%)

© 2021 Merlin S et al. *JAMA Network Open.*

>2 Years	189 (48.3%)
No Response	6 (1.5%)
**Length of time in current position**	
Less Than 6 Months	25 (6.4%)
6 Months-1 Year	111 (28.4%)
1-2 Years	106 (27.1%)
>2 Years	148 (37.9%)
No Response	1 (0.3%)
**Sales commission**	
No	353 (90.3%)
Yes	36 (9.2%)
No Response	2 (0.5%)
**Education**	
Completed high school/GED or less	46 (11.8%)
Some college or Associates Degree	164 (41.9%)
Completed 4-year college degree	111 (28.4%)
Some graduate school	18 (4.6%)
Completed graduate school	49 (12.5%)
Prefer not to answer	2 (0.5%)
No Response	1 (0.3%)
**Gender**	
Male	172 (44%)
Female	206 (52.7%)
Other/No response	13 (3.3%)
**State medicalization score**	47.9 ± 15.4
**Statewide adult use**	
No	254 (65%)
Yes	137 (35%)

© 2021 Merlin S et al. *JAMA Network Open.*

eTable 2. Sensitivity Analysis (N=391): Self-report of basis of recommendations

Basis of recommendation	Yes
Customer's medical condition(s)	300 (76.7%)
Experiences of other customers	295 (75.4%)
Customer's prior experience with cannabis	286 (73.1%)
Customer preference	281 (71.9%)
Daytime or nighttime consumption	280 (71.6%)
Scientific articles (e.g. articles from medical journals)	271 (69.3%)
Your personal experience	266 (68%)
Training provided by your employer	253 (64.7%)
Other staff recommendations	236 (60.4%)
Product availability	201 (51.4%)
Cost	192 (49.1%)
Experience of friends or colleagues	184 (47.1%)
Trade literature (e.g. trade magazines or websites)	169 (43.2%)
Physician/clinician input	165 (42.2%)
App or website that helps with product selection (e.g. Strainpaint)	131 (33.5%)
Product smell	122 (31.2%)
Product appearance (for flower)	120 (30.7%)
What needs to get moved out of inventory	40 (10.2%)

© 2021 Merlin S et al. *JAMA Network Open.*

eTable 3. Sensitivity Analysis (N=391): Association of basis of recommendations with state medicalization score and statewide adult use*

Potential Basis	State medicalization score (per 10 point increment)			Statewide adult use		
	Odds Ratio	95% CI	P-value	Odds Ratio	95% CI	P-value
Training provided by your employer	1.38	(1.15, 1.66)	<0.01	1.48	(0.86, 2.55)	0.16
Trade literature (e.g. trade magazines or websites)	0.93	(0.79, 1.1)	0.40	2.0	(1.2, 3.35)	0.01
App or website that helps with product selection (e.g. Strainpaint)	0.89	(0.75, 1.06)	0.18	1.67	(0.99, 2.82)	0.05
Scientific articles (e.g. articles from medical journals)	0.84	(0.71, 0.99)	0.04	1.47	(0.84, 2.58)	0.18
Physician/clinician input	1.27	(1.08, 1.49)	<0.01	0.81	(0.48, 1.36)	0.43
Customer's medical condition(s)	0.97	(0.80, 1.18)	0.78	0.73	(0.4, 1.35)	0.31
Cost	1.0	(0.85, 1.17)	0.96	1.15	(0.69, 1.90)	0.59
Product availability	0.91	(0.78, 1.07)	0.26	1.08	(0.66, 1.79)	0.75
What needs to get moved out of inventory	0.77	(0.58, 1.03)	0.08	1.09	(0.51, 2.33)	0.83
Experiences of other customers	0.98	(0.81, 1.18)	0.81	2.03	(1.06, 3.9)	0.03
Your personal experience	0.79	(0.66, 0.95)	0.01	1.59	(0.81, 3.11)	0.18
Other staff recommendations	0.82	(0.69, 0.97)	0.02	1.61	(0.90, 2.88)	0.11
Customer preference	1.0	(0.83, 1.19)	0.96	1.17	(0.65, 2.11)	0.6
Experience of friends or colleagues	0.85	(0.72, 1.01)	0.07	2.71	(1.58, 4.64)	<0.01
Customer's prior experience with cannabis	0.99	(0.82, 1.2)	0.92	1.81	(0.95, 3.45)	0.08
Daytime or nighttime consumption	0.91	(0.75, 1.1)	0.33	1.98	(0.99, 3.95)	0.05
Product smell	0.87	(0.72, 1.06)	0.17	3.33	(1.92, 5.79)	<0.01
Product appearance (for flower)	0.77	(0.63, 0.95)	0.01	2.97	(1.70, 5.17)	<0.01

*This table presents a series of logistic regression models in which each row represents the dependent variable with each column representing an independent variable in separate logistic regression models. For example, the top-left-hand cell indicates that a 10-point increase in the state medicalization score is associated with 1.41 times higher odds of the respondent saying that they use training provided by their employer as basis for recommendations

© 2021 Merlin S et al. *JAMA Network Open.*

eTable 4. Sensitivity Analysis (N=391): How often respondent talks to customers about risk

Risk	1 - Never	2	3	4	5 - Always
Cannabis use disorder/addiction	119 (30.4%)	111 (28.4%)	88 (22.5%)	51 (13%)	21 (5.4%)
Motor vehicle accidents/safe driving	65 (16.6%)	91 (23.3%)	96 (24.6%)	75 (19.2%)	63 (16.1%)
Cannabis withdrawal symptoms	140 (35.8%)	114 (29.2%)	71 (18.2%)	46 (11.8%)	19 (4.9%)
Psychotic reaction	102 (26.1%)	88 (22.5%)	89 (22.8%)	58 (14.8%)	53 (13.6%)
Cannabis medication interactions	59 (15.1%)	75 (19.2%)	81 (20.7%)	88 (22.5%)	87 (22.3%)
Potential cannabis side effects (e.g. sleepiness, paranoia)	14 (3.6%)	35 (9%)	69 (17.6%)	111 (28.4%)	160 (40.9%)
Safe storage away from children and pets	18 (4.6%)	38 (9.7%)	73 (18.7%)	83 (21.2%)	178 (45.5%)

© 2021 Merlin S et al. *JAMA Network Open.*

eTable 5. Sensitivity analysis (N=391): Association of talking to customers about risk with state medicalization score and statewide adult use*

Risk (outcome)	State medicalization score (per 10 point increment)		Statewide adult use	
	B (SE)	P-value	B (SE)	P-value
Cannabis use disorder/addiction	<0.01(0.04)	0.85	-0.24 (0.14)	0.08
Motor vehicle accidents/safe driving	0.02 (0.05)	0.7	0.01 (0.16)	0.96
Cannabis withdrawal symptoms	-0.02 (0.04)	0.68	-0.27 (0.14)	0.05
Psychotic reaction	0.06 (0.05)	0.28	-0.27 (0.16)	0.1
Cannabis medication interactions	0.08 (0.05)	0.12	-0.19 (0.16)	0.26
Potential cannabis side effects (e.g. sleepiness, paranoia)	-0.01 (0.04)	0.89	-0.21 (0.14)	0.13
Safe storage away from children and pets	0.02 (0.04)	0.67	0.29 (0.14)	0.05

*This table presents a series of linear regression models in which each row represents the dependent variable with each column representing an independent variable in separate linear regression models. For example, the top-left-hand cell indicates that a 10-point increase in the state medicalization score is associated with a mean change of 0.003 in the scale response to how often the respondent talks to their customers about the risks of cannabis use disorder/addiction.

© 2021 Merlin S et al. *JAMA Network Open.*

eFigure1. Survey response by state, primary analysis, N=434*

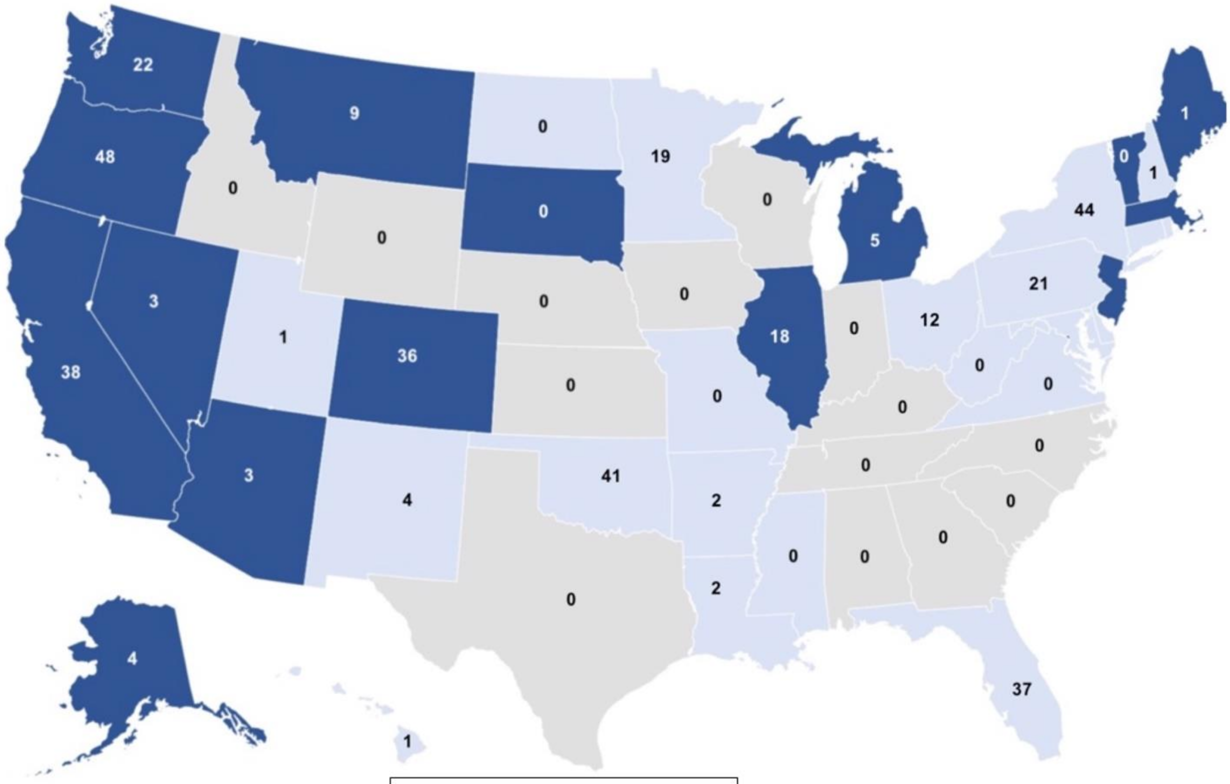

State	Freq	State	Freq
Missing	2	ME	1
AK	4	MI	5
AR	2	MN	19
AZ	3	MT	9
CA	38	NH	1
CO	36	NJ	3
CT	5	NM	4
DC	4	NV	3
DE	9	NY	44
FL	37	OH	12
HI	1	OK	41
IL	18	OR	48
LA	2	PA	21
MA	8	RI	4
MD	27	UT	1
ME	1	WA	22

*States where all cannabis is illegal are shaded in grey, states where only medical cannabis is legal are shaded in light blue, and states where cannabis is legal for both medical and adult use are shaded in dark blue.

© 2021 Merlin S et al. *JAMA Network Open.*

eFigure 2. Sensitivity Analysis: Survey response by state, N=391*

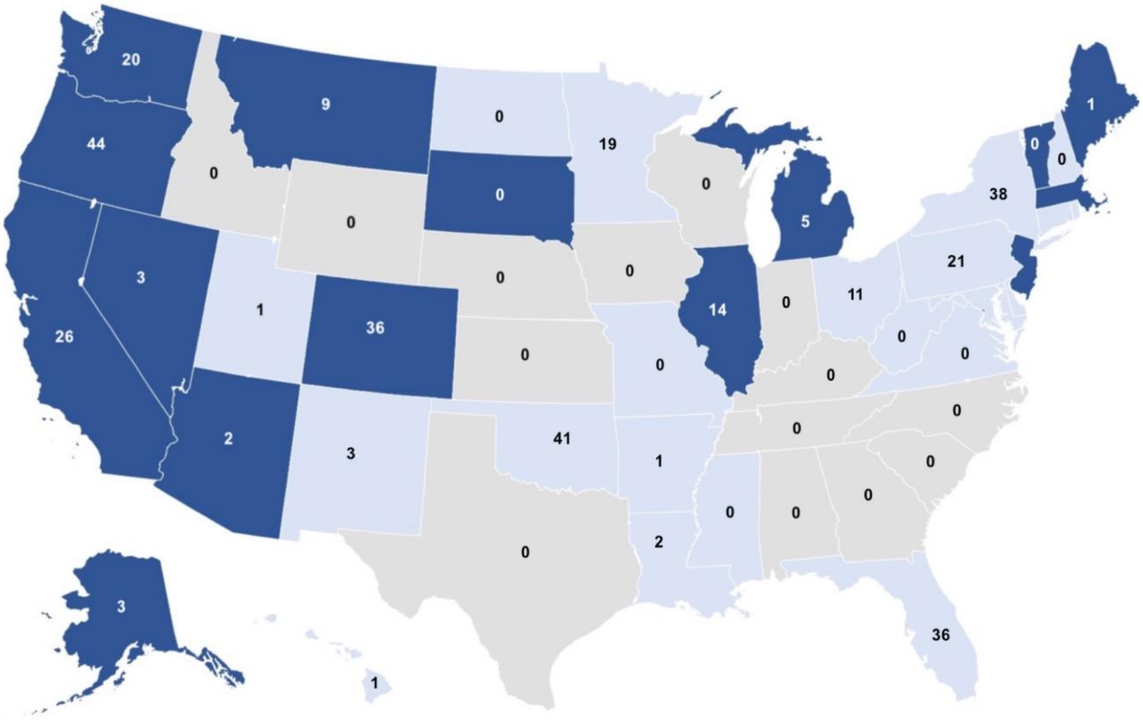

State	Freq	State	Freq
Missing	1	ME	5
AK	3	MI	19
AR	1	MN	9
AZ	2	MT	5
CA	26	NJ	1
CO	36	NM	3
CT	4	NV	3
DC	1	NY	38
DE	9	OH	11
FL	36	OK	41
HI	1	OR	44
IL	14	PA	21
LA	2	RI	4
MA	7	UT	1
MD	27	WA	20
ME	1		

*States where all cannabis is illegal are shaded in grey, states where only medical cannabis is legal are shaded in light blue, and states where cannabis is legal for both medical and adult use are shaded in dark blue.

© 2021 Merlin S et al. *JAMA Network Open.*
